# Effect of garcinol against arsenic-induced neurobehavioral alterations and liver and kidney dysfunction in albino mice

**DOI:** 10.37796/2211-8039.1650

**Published:** 2025-09-01

**Authors:** Abdulmohsen I. Algefare, Manal A. Alfwuaires

**Affiliations:** Department of Biological Sciences, College of Science, King Faisal University, Al-Ahsa 31982, Saudi Arabia

**Keywords:** Arsenic, Brain, Garcinol, Oxidative stress, Nrf2

## Abstract

Prolonged exposure to inorganic arsenic is commonly linked to brain damage via oxidative and apoptotic processes. The compound garcinol (GCL) has garnered significant interest because of its beneficial effects on human health. However, the protective ability of GCL against arsenic-induced toxicity in the brain remains unexplored. Therefore, our study aimed to examine the neuroprotective effect of GCL against the adverse impact of sodium arsenite (SA) on behavioral patterns, molecular mechanisms, apoptotic markers, and oxidative stress parameters in the brain, liver, and kidneys of mice. The mice were categorized into four distinct groups for 28 days: Group I, referred to as the Control group, received a 5 % v/v solution of dimethyl sulfoxide (DMSO); Group II, referred to as the SA group, received a dosage of 20 mg/kg of SA; Group III, referred to as the SA + GCL group, received a combined dosage of 20 mg/kg of SA and 50 mg/kg of GCL; and Group IV, referred to as the GCL group, received a dosage of 50 mg/kg of GCL. Following drug administration, the behavior of the animals was evaluated and analyzed. Additionally, the levels of acetylcholinesterase (AChE), ATP hydrolysis, and angiotensin I-converting enzyme (ACE), which are associated with cognitive function, were examined. Our study demonstrated that the administration of GCL enhanced cognitive behavior. Additionally, GCL mitigated cholinergic deficits as evidenced by a reduction in AChE activity. Furthermore, GCL increased the signaling of glycogen synthase kinase 3 beta (GSK3β) and cAMP response element-binding protein (CREB) in SA-treated mice, enhanced redox equilibrium, and protected against oxidative damage caused by SA in the brains of mice. This effect was mediated by the activation of nuclear factor erythroid 2-related factor 2 (NRF2)/heme oxygenase-1 (HO-1) proteins, resulting in a significant decrease in malondialdehyde concentration. Thus, this preclinical study showed that treatment with GCL ameliorated neurobehavior, modulated cognitive function-associated biomarkers, and protected mice from SA-induced neurotoxicity.

## 1. Introduction

There is widespread contamination of groundwater in many developing countries by arsenic, a carcinogenic and toxic metalloid [1]. The increased presence of arsenic in the environment significantly affects the well-being of aquatic organisms. The bioavailability of arsenic in sediments results in its uptake by benthic fish via dietary routes [2]. According to the World Health Organization (WHO), the acceptable threshold for arsenic concentration in drinking water is 10 parts per billion (ppb) [3]. Abdominal discomfort, severe diarrhea, and vomiting are recognized symptoms associated with acute arsenic intoxication [4,5]. Long-term use of arsenic leads to the build-up of this toxic substance in vital body organs, which subsequently causes hypertension, atherosclerosis, and peripheral nerve impairment [6,7]. The susceptibility of liver and kidneys to poisoning is attributed to their crucial roles in metabolic and excretory functions. Absorption of arsenic occurs predominantly in the small intestine, after which it is distributed to numerous organs, including the liver. Arsenic accumulation occurs in the liver under continuous and prolonged exposure. Chronic arsenic exposure has been associated with the development of liver fibrosis, liver cirrhosis, and hepatomegaly [8,9]. Renal function converts pentavalent arsenic into its hazardous trivalent form, which exhibits reduced solubility resulting in potential harm to tubular structures, glomerular units, and capillary networks [10].

Multiple epidemiological studies have demonstrated a positive association between elevated arsenic levels in drinking water and the occurrence of neurological and behavioral abnormalities, including reduced locomotor activity, altered cognitive functioning, and prenatal difficulties [11,12]. The ability of arsenic to readily traverse the blood–brain barrier and accumulate within several regions of the brain, including the striatum and hippocampus, exacerbates its toxic effects and damages the affected tissues [6,11]. Arsenic exposure also impacts cognitive function. The cytoskeletal organization is compromised, resulting in detrimental effects on the axons. Arsenic exhibits an affinity for sulfhydryl groups, leading to the formation of reactive oxygen species (ROS), which subsequently induces cell death in the exposed organism [13]. Nuclear factor erythroid 2-related factor 2 (Nrf2) plays a central role in the modulation of oxidative stress through its ability to activate transcription. This activation subsequently upregulates the production of antioxidant enzymes and regulates the expression of genes relevant to antioxidants, including heme oxygenase 1 (HO-1) [14]. According to Rajendran et al. (2022), an increase in oxidative stress or damage in albino mice results in pronounced vulnerability to oxidative damage and decreased production of Nrf2 in Nrf2 mutant mice. This suggests that Nrf2 has neuroprotective properties [15]. Furthermore, glycogen synthase kinase beta (GSKβ) and cAMP response element-binding protein (CREB) signaling pathways are activated, which have an important role in learning, memory, and cytoprotection [16]. Earlier, dimercaprol was the most commonly prescribed chelating agent for arsenic. Dimercaprol has been associated with several adverse side effects, including diaphoresis, lacrimation, blepharospasm, rhinorrhea, nausea, vomiting, stomach pain, tachycardia, hypertension, headache, and burning sensations in the mouth, nose, and eyes. While it is known that the brain system is susceptible to arsenic, the effects of arsenic on crucial aspects of brain transmission remain unclear. Given the ongoing exposure of a substantial portion of the population to arsenic and the resulting risk of central nervous system toxicity, there is considerable concern regarding the ability to safeguard against arsenic-induced brain impairment. Therefore, it is necessary to investigate potential therapeutic strategies to alleviate the neurotoxic effects of arsenic. Evidence suggests that natural plant products have significant potential for preventing or reducing arsenic-induced neurotoxicity [17,18].

Garcinol (GCL) is a member of the polyisoprenylated benzophenone family, and is derived from the fruits and leaves of many Garcinia species, including Garcinia indica. This species is a tiny evergreen tree indigenous to tropical regions of Asia and Africa [16,19]. The desiccated pericarp of G. indica, often known as kokum, is employed as a culinary embellishment in curry dishes, and holds a significant place in traditional Indian medicine because of its therapeutic properties in managing inflammatory and infectious ailments. Prior studies have substantiated the anti-inflammatory, antioxidative, and anticancer properties of GCL that contribute to its potential therapeutic effects [19–21]. Nevertheless, there is a lack of research on the impact of GCL on biomolecules related to cognitive function in mice treated with sodium arsenite (SA). Nrf2 regulates cellular redox homeostasis by acting as a transcription factor. It alleviates oxidative stress by controlling antioxidant response element (ARE) genes, including HO-1, glutathione (GSH) production enzymes, superoxide dismutase (SOD), and glutathione peroxidase (GPx) [22]. Nrf2 function is regulated by upstream regulators, including GSK3β. Activated GSK3β negatively regulates Nrf2 by directly phosphorylating and degrading it. Activated GSK3β phosphorylates Fyn kinase, which ultimately phosphorylates Nrf2, thereby causing its translocation from the nucleus to cytosol and the destruction of proteasomes. GSK3β is inactivated by the phosphorylation of its serine 9 residue by protein kinase B. This eliminates the inhibitory effect of GSK3β on Nrf2 [23]. Thus, Nrf2 degradation decreases, and nuclear translocation increases with the expression of ARE genes, such as HO-1, a redox-sensitive enzyme that transforms heme into biliverdin. Anti-inflammatory and antioxidant properties of Nrf2 also inhibits cell death. It boosts Bcl-2 gene transcription and protein levels, which reduces Bax expression and apoptosis [24]. Additionally, GSK-3β upstream of Fyn kinase phosphorylates Nrf2 tyrosine 568, causing nuclear export of Nrf2. Inhibitory phosphorylation of GSK-3β may cause nuclear accumulation of Nrf2 [25]. Various studies have reported that GCL activates Nrf2, which leads to increased expression of HO-1 [26,27]. The activation of Nrf2/HO-1 is believed to contribute to the antioxidant and anti-inflammatory effects of GCL [28,29]. Studies have shown that GCL can hinder oxidative stress and enhance the damage caused by cerebral ischemia [30]. This work examined the activation of GSK3β/Nrf2/HO-1 pathway by GCL protects the liver, kidney, and brain in mice following SA-induced damage.

## 2. Methods

### 2.1. Reagents

Sigma Chemical Co. (St. Louis, MO, USA) supplied all the reagents including SA (7784-46-5), and GCL (GC13474) was obtained from GlpBio.

### 2.2. Animals

The study included male Swiss albino mice that were 6–8 weeks old and weighed 25–30 g. These mice were housed in plastic cages under controlled conditions, including a temperature of 22 ± 2 °C, humidity of 50 ± 10 %, and a 12-h light/dark cycle. The mice were provided with unrestricted access to a normal balanced rodent meal and water. The animals were acclimatized for 1 week prior to their use in experimental procedures conducted at the Department of Biological Sciences, King Faisal University in Al Ahsa, Saudi Arabia. The study was conducted according to the guidelines of King Faisal University and the “Executive Regulations for Research Ethics on Living Creatures (Second Edition),” published by the National Bioethics Committee, Saudi Arabia. All animal care and experimental procedures were approved by the Animal Research Ethics Committee of the King Faisal University Declaration of King Faisal University and the Institutional Review Board (KFUREC-2022-APR-EA0003290).

### 2.3. Animal experiment

Following the acclimation phase, the mice were randomly divided into four distinct groups, each consisting of six animals, as outlined below: Group I, referred to as the Control group, received a 5 % v/v solution of DMSO; Group II, referred to as the SA group, received a dosage of 20 mg/kg of SA [31,32]; Group III, referred to as the SA + GCL group, received a combined dosage of 20 mg/kg of SA and 50 mg/kg of GCL; and Group IV, referred to as the GCL group, received a dosage of 50 mg/kg of GCL [33–35]. Following the conclusion of the experimental period, and after a duration of 24 h subsequent to the administration of the final medication, the mice were humanely euthanized through intraperitoneal injection of pentobarbital at a dosage of 45 mg/kg. The brains were carefully removed, rinsed with cold isotonic saline solution to eliminate any traces of blood, and immersed in a 4 % neutral-buffered formalin solution for fixation. The cells were then subjected to histological analysis and immunoblotting. The remaining portion was preserved at a temperature of −80 °C for future research.

### 2.4. Morris water maze (MWM) test

This experiment followed the previous protocol published by Barai et al. (2018) and Akomolafe et al. (2020) [36,37]. The MWM test was performed using a circular tank with a diameter of 120 cm and a height of 50 cm. The pool of water in the tank had a depth of 30 cm. The water temperature was controlled at 27 ± 2 °C, and the maze was partitioned into four equidistant quadrants labeled as North (N), East (E), South (S), and West (W). A tiny rectangular concealed platform was consistently placed in the southeastern quadrant during the experiments. Throughout the evaluation period, the platform was maintained in a submerged state and positioned 1 cm below the water surface. The duration (in seconds) required to identify the concealed platform (for escaping from the water) was measured to evaluate the performance.

### 2.5. Y-maze test

The experiment followed the protocol described by Akinyemi et al. (2017b) [38]. The Y-maze test was used to assess cognitive function in the experimental rodents. The experimental setup consisted of three identical arms, each 40 cm long, 40 cm high, and 15 cm wide. The arms were designated as X, Y, and Z and arranged at equidistant angles. Each rat was positioned at the center and allowed unrestricted movement to select an arm for a test period of 5 min. Arm entries were recorded using computerized technology. Alternations, characterized by successive entries in overlapping triplet sets (XYZ, YZX, and ZXY), were designated as alternations. Alternation frequencies between the arms were recorded and plotted to ascertain the memory index, which was expressed as the percentage of alternation.

### 2.6. Arsenic measurement in brain tissue

The method developed by Cui et al. was used to estimate the levels of arsenic in the cortical tissues of mice [18,39]. In brief, 0.5 g of cortical tissue was digested in a solution consisting of a mixture of HNO3-HCLO4 for a duration of 48 h at a temperature of 130 °C. Following the evaporation of HNO3, the digested sample was further diluted with deionized water. The concentration of arsenic was evaluated using an atomic absorption spectrophotometer equipped with a graphite furnace.

### 2.7. Assays for acetylcholinesterase (AChE) activities

AChE activity was measured in accordance with the methodology outlined in a previous study [15]. A solution was prepared by combining phosphate buffer with a concentration of 100 mmol/L and a pH of 7.5, along with 5,5-dithiobisnitrobenzoic acid at a concentration of 1 mmol/L. The enzyme was supplemented with 200 μL of the prepared solution, while the enzyme itself contained 30–40 μg of protein. After 120 s, acetylthiocholine iodide (at a concentration of 0.8 mmol/l) was added to commence the reaction. AChE activity was quantified as micromoles of acetylthiocholine hydrolyzed per hour per milligram of protein.

### 2.8. The determination of angiotensin-converting enzyme (ACE) activity

ACE activity in brain homogenates was quantified using the methodology developed by Cushman and Cheung [40], which involves quantifying the amount of hippuric acid generated from hippurylhistidyl-leucine. A solution of hippurylhistidylleucine (Bz-Gly-His-Leu) at a concentration of 8.33 mM in a volume of 150 μL was combined with tissue homogenates and Tris–HCl buffer at a concentration of 125 mM and a pH of 8.3. The solution was incubated at a temperature of 37 °C for 30 min. Hydrochloric acid (1 M, 250 μL) was then introduced into the solution to terminate the ongoing reaction. The generated hippuric acid was solubilized in a solution of ethyl acetate (1.5 mL). The solution was centrifuged, and 1 mL of the ethyl acetate layer was transferred to a test tube and evaporated. The residue was dissolved in water and the absorbance was recorded at 228 nm. ACE activity was quantified and reported as millimoles per milligram of protein.

### 2.9. Measurement of ATP hydrolysis

Assays for ectonucleoside triphosphate diphosphohydrolases (E-NTPDase) and ecto-5′-nucleotidase in brain tissues were performed according to a previously published method [41]. The reaction mixture was composed of 112.5 mM Tris–HCl at pH 8.0, water, and 1.0 mg protein. During the pre-incubation phase, a 10 min period was allocated for the addition of the extract. This process took place in a water bath maintained at a temperature of 37 °C, before the introduction of the substrate. To initiate the reactions, 3.0 mM of ATP substrates was introduced into a reaction chamber of 200 μL. Following a 40 min incubation period, the enzymatic processes were halted by the addition of 200 μL of trichloroacetic acid (TCA) at a final concentration of 5 %. Incubation periods and protein concentrations were selected to ensure linearity of the enzymatic reactions. Inorganic phosphate (Pi) was quantified using a colorimetric technique, as described in a previous study [37]. A spectrophotometer was used with the wavelength set at 630 nm. Controls were included to account for non-enzymatic substrate hydrolysis by introducing brain tissue subsequent to the cessation of the reaction using TCA. The experimental procedure was conducted in duplicate for all the samples. Enzyme activity is commonly denoted as the release of nanomoles of inorganic phosphate per minute per milligram of protein.

### 2.10. Liver marker enzymes

Serum activity of hepatic function biomarkers was evaluated. Aspartate aminotransferase (AST) and alanine transaminase (ALT) levels were measured using commercial kits (Randox Laboratories Ltd., Crumlin, UK).

### 2.11. Measurement of renal biochemical marker

Serum blood urea nitrogen (BUN) and creatinine (CREA) levels were determined. A small amount of blood (approximately 0.5 mL) was withdrawn from the retro-orbital plexus. A Sekisui Medical (Tokyo, Japan) assay kit was used to measure BUN and CREA levels.

### 2.12. Antioxidant enzymes

The mouse brain samples were assayed for oxidative stress. Protein concentration was measured according to the Bradford assay [42], while the SOD and malondialdehyde (MDA) activities were evaluated using a previously reported method [43].

### 2.13. Western blot analysis

Western blot analysis was performed as previously described [44]. A 3600-00-C-Digit Blot Scanner was used to visualize protein bands. The control band was normalized to 1 using Image Studio Lite software (Lincoln, NE, USA). Each experiment was performed in triplicate.

### 2.14. Histopathology

The brain tissues were embedded in paraffin, cut into 4 μm thick sections, stained with hematoxylin (H&E), and were then fixed in 10 % formalin. An optical microscope (Leica D6000; Leica, Wetzlar, Germany) was used to observe and measure the prepared sections, and images were acquired (200 × magnification).

### 2.15. Statistical analysis

Data are represented as means ± standard error of mean (SEM) and were analyzed using Prism 5.0 statistical program (GraphPad Software Inc., San Diego, CA, USA). Comparisons between experimental groups were performed using one-way analysis of variance (ANOVA) followed by Tukey’s post-hoc test. Differences were considered significant if the p-value was <0.05. *p < 0.05 indicates significant differences between the SA and control groups. #p < 0.05 indicates significant differences between the SA and SA + GCL groups.

## 3. Results

### 3.1. Effect of GCL on mice survival and organ weight

**Fig. 1:**
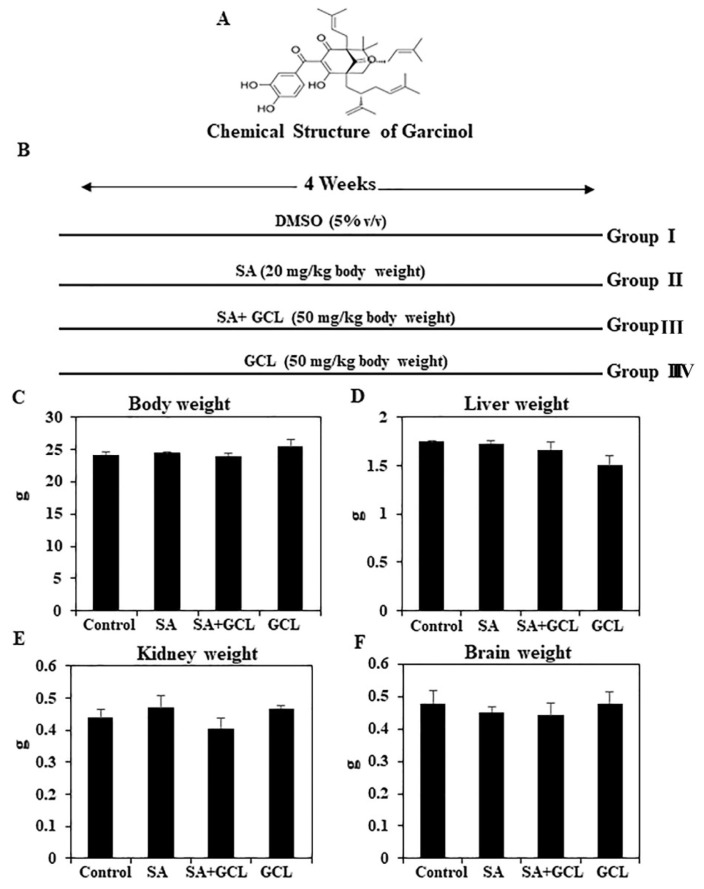
Effect of SA, GCL, and their co-treatment on body and organ weights. (A). Chemical structure of GCL. (B) Graphical abstract of animal experiment. (C) Body weight (g). (D) Liver weight (g). (E) Kidney weight (F) Brain weight. Differences were considered significant if the p-value was <0.05. *p < 0.05 indicates significant differences between the SA and control groups. #p < 0.05 indicates significant differences between the SA and SA + GCL groups.

There were no noticeable differences in the appearance of the 24 mice (6 per group) that remained alive at the conclusion of the treatment period. Mice were divided into four distinct experimental groups. Fig. 1A shows the chemical structure of GCL, and Fig. 1B provides a graphical representation of the animal experimental study. Toxicity testing on animals is necessary for evaluating drug, chemical, biological, food additive, and medical device safety. When evaluating the impact of novel compounds or other chemicals on chemically stimulated organs, it is routine practice to quantify changes in body and organ weight [45]. In the current study, there was no significant difference in body, kidney, or brain weights between the control and treated groups (Fig. 1C, D, E, and F).

### 3.2. Effects of GCL on SA-induced cognitive impairment

**Fig. 2:**
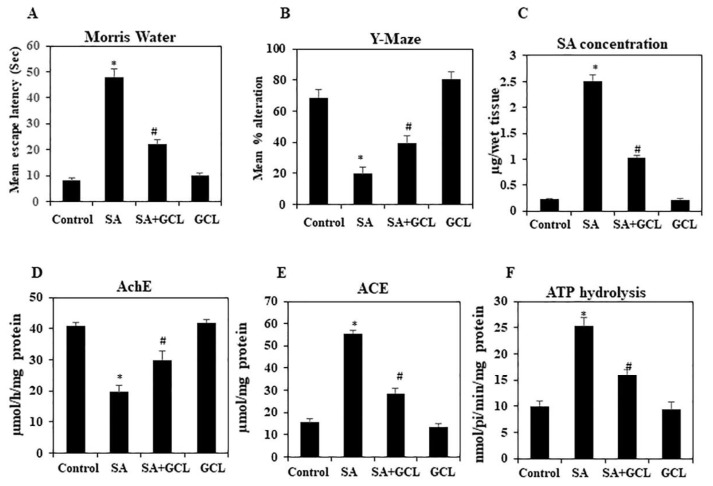
Effect of GCL on alternation behavior and escape latency in SA-treated mice. (A) Morris water test. (B) Y-maze test. (C) Concentration of SA in brain tissue. (D) AChE activity (E) ACE activity (F) ATP hydrolysis activity. Differences were considered significant if the p-value was <0.05. *p < 0.05 indicates significant differences between the SA and control groups. #p < 0.05 indicates significant differences between the SA and SA + GCL groups.

The MWM test is a spatial learning test designed for laboratory animals. It involves the use of distal signals to guide rodents as they travel from various starting points around the edge of a swimming arena to find a submerged escape platform. The evaluation of spatial learning involves multiple iterations, and the establishment of a reference memory is ascertained by observing the inclination towards the platform region in the absence of the platform. The implementation of reversal and shift trials augmented the identification of spatial deficits [46]. Fig. 2A depicts the use of MWM test to assess the impact of GCL on cognitive impairment induced by SA in mice. Sodium arsenite resulted in a significant increase in escape latency compared to the control group. However, a decrease in escape latency was observed after treatment with GCL in combination with SA.

Fig. 2B illustrates the effects of SA on the spontaneous alternation behavior of mice with and without GCL treatment. The implementation of SA resulted in a statistically significant decrease (p < 0.05) in the proportion of alternations compared to the control group. The administration of GCL resulted in a statistically significant improvement (p < 0.05) in the altered behavior induced by SA, as depicted in Fig. 2B.

### 3.3. GCL affects SA-induced brain arsenic levels

As anticipated, the concentration of arsenic in the brains of mice in Group II was notably higher than that in the brains of mice in Group I. In contrast, the concentration of arsenic in the brain tissue of mice in Group III (GCL + SA) was much lower, as shown in Fig. 2C.

### 3.4. GCL affects AChE activity in brain tissue

To investigate the potential of GCL in enhancing AChE activity, mice were administered GCL. Fig. 2D illustrates that the administration of SA resulted in a notable reduction in AChE activity in cortical tissue. However, in the cohort of mice that received GCL prior to SA treatment, AChE activity was considerably increased compared to that in Group III.

### 3.5. Effect of GCL on ACE activity in brain tissue

The administration of GCL did not have a significant impact on ACE activity in the GCL group, as shown in Fig. 2E. However, the administration of SA resulted in a significant elevation in ACE activity compared to that in the control groups. A notable reduction in ACE activity was observed in brain tissue in the SA group when treated with GCL.

### 3.6. Effect of GCL on ATP hydrolysis activity in brain tissue

NTPDase activity in the mouse brain in the GCL group, in the presence of ATP did not exhibit a significant difference compared to that in the control group, as depicted in Fig. 2F. Nevertheless, the administration of SA resulted in a significant increase in NTPDase activity in the SA group compared to that in the control group. Moreover, Group III exhibited decreased NTPDase activity following GCL administration.

### 3.7. GCL liver marker enzymes in SA-induced mice

**Fig. 3:**
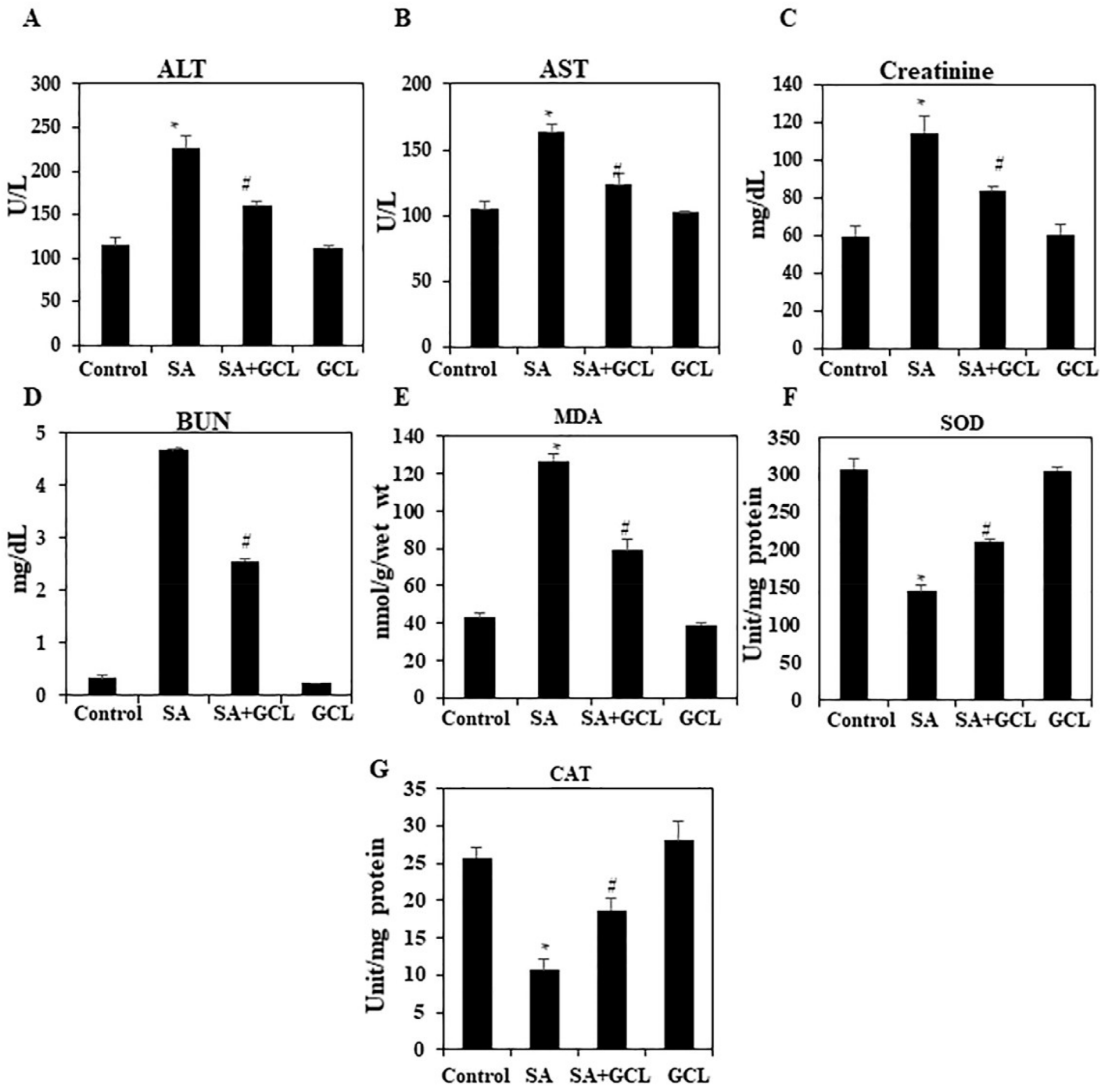
Effect of SA, GCL, and their co-treatment on the activity of liver and kidney maker enzymes. (A) ALT (serum). (B) AST (serum). (C) Creatinine (serum). (D) Blood urea (serum). (E) MDA level (liver tissue). (F) SOD activity (liver tissue). (G) CAT (liver tissue) activity. Differences were considered significant if the p-value was <0.05. *p < 0.05 indicates significant differences between the SA and control groups. #p < 0.05 indicates significant differences between the SA and SA + GCL groups.

Administration of SA resulted in a significant increase in serum enzyme levels compared to the control group, suggesting the presence of toxicity. The experimental group of animals that received SA + GCL exhibited a noteworthy reduction in serum ALT and AST levels (Fig. 3A and B) compared to the group that was treated with SA alone.

### 3.8. Effect of GCL on kidney marker enzymes in SA-induced mice

Elevated serum levels of kidney enzymes, specifically creatinine and urea, were detected in SA-treated animals. However, when the group treated with SA + GCL was compared to the group treated with SA alone, a statistically significant decrease was observed (Fig. 3C and D).

### 3.9. Effect of GCL on antioxidant enzymes in the liver

The mice in Group II exhibited notably elevated levels of lipid peroxidation (LPO) following exposure to arsenic compared to the animals in Group I (Fig. 3E). However, the levels of LPO in the brain tissue of animals in Group III (GCL + SA) were much lower. The activities of antioxidant enzymes, specifically SOD and catalase (CAT), were considerably lower in Group II mice than in Group I mice (Fig. 3F and G). Nevertheless, the levels of oxidative stress markers were significantly restored in Group III mice (GCL + SA).

### 3.10. Effect of GCL on Nrf2 and HO-I expression in SA-induced mice

**Fig. 4:**
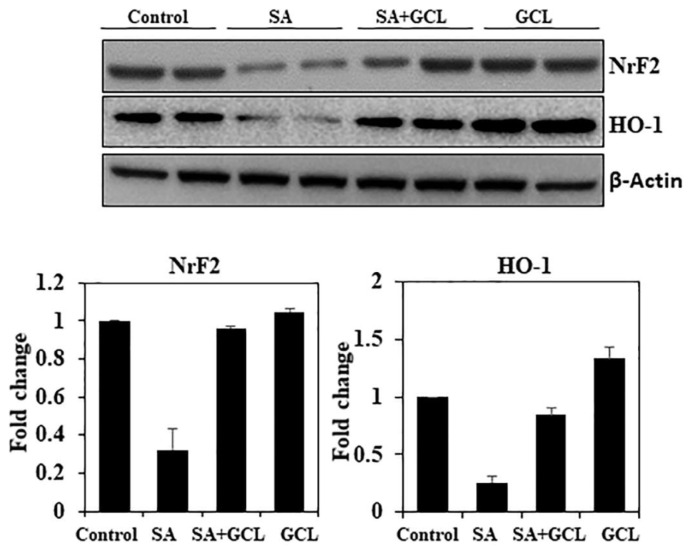
Effect of SA, GCL, and their co-treatment on the activity of Nrf2 and HO-1 in brain tissue. Differences were considered significant if the p-value was <0.05. *p < 0.05 indicates significant differences between the SA and control groups. #p < 0.05 indicates significant differences between the SA and SA + GCL groups.

Activation of the cis-acting antioxidant response element (ARE) relies on the transcription factor Nrf2. Nrf2 is responsible for the activation of various antioxidant genes that play crucial roles in mitigating oxidative stress in biological systems [47]. One such gene is hemeoxygenase-1 (HO-1). Given the observed cytoprotective, antioxidant, and anti-inflammatory capabilities of these enzymes, we initially hypothesized that GCL inhibits SA-induced oxidative stress and apoptosis through the activation of antioxidant genes, including HO-1 and its transcriptional activator Nrf2 (Fig. 4). The findings of our investigation provide empirical support for our theory, as they demonstrate a considerable increase in the expression of HO-1 and Nrf2 following the administration of GCL.

### 3.11. Effect of GCL on pGSK3β and pCREB expression in SA-induced mice

**Fig. 5:**
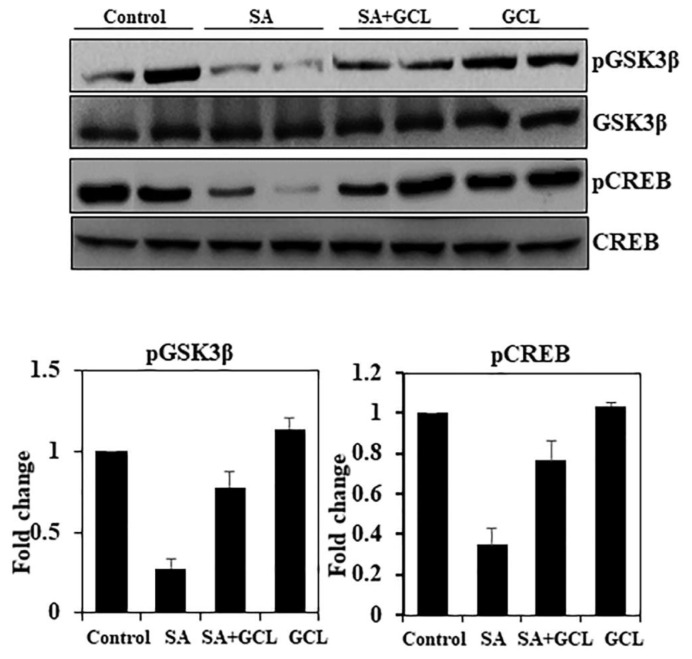
Effect of SA, GCL, and their co-treatment on the activity of pGSK3β/pCREB in brain tissue. Differences were considered significant if the p-value was <0.05. *p < 0.05 indicates significant differences between the SA and control groups. #p < 0.05 indicates significant differences between the SA and SA + GCL groups.

The participation of CREB, a transcription factor, was further evaluated to gain a deeper understanding of its role in arsenic-induced toxicity. An observable reduction in the levels of pGSK3β/GSK3β and pCREB/CREB was detected upon exposure to arsenic (Fig. 5). Notably, concurrent exposure to GCL provided a safeguard against alterations caused by arsenic in brain tissue. Nevertheless, there was no notable alteration in the levels of pGSK3β/GSK3β and pCREB/CREB in mice administered with GCL compared to those in the control group.

### 3.12. Effect of GCL on Bcl-2 and cleaved caspase-3 expression in SA-induced mice

**Fig. 6:**
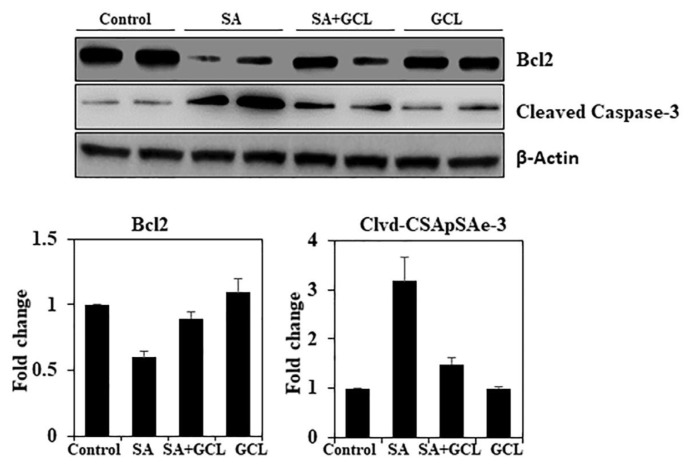
Effect of SA, GCL, and their co-treatment on the activity of Bcl-2 and cleaved caspase-3 in the brain tissue. Differences were considered significant if the p-value was <0.05. *p < 0.05 indicates significant differences between the SA and control groups. #p < 0.05 indicates significant differences between the SA and SA + GCL groups.

Caspase-3 plays a significant role as an effector molecule, influencing key apoptotic processes such as chromatin condensation, DNA fragmentation, and the production of apoptotic bodies. Consequently, we hypothesized that SA-induced DNA damage and apoptosis are facilitated by the activation of caspase-3. According to the findings presented in Fig. 6, the administration of SA resulted in a considerable increase in caspase-3 activation in brain tissue. Conversely, GCL administration notably inhibited caspase-3 activation. The expression of Bax, a protein that promotes programmed cell death, is increased by exposure to toxins, whereas that of Bcl-2, a protein that inhibits programmed cell death, is reduced. Consistent with this finding, SA exposure significantly reduced Bcl-2 expression in Fig. 6. In contrast, pre-treatment with GCL resulted in the upregulation of Bcl-2 in nerve tissues. The data presented in this study provide compelling evidence that the neuroprotective effect of GCL against SA-induced apoptosis is attributable to the activation of caspase-3 and the dysregulation of Bcl-2.

### 3.13. Histopathological analysis

**Fig. 7:**
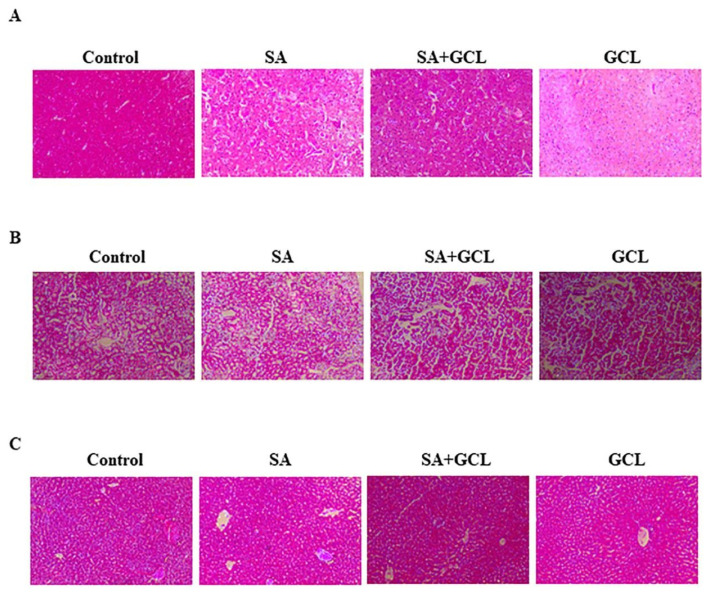
Effect of SA, GCL, and their co-treatment on tissue morphology using H&E (400×) staining. (A) Brain tissue (B) Kidney (C) Liver.

The brains of control mice had a consistent and typical organization and morphology of cerebral capillaries (referred to as capillary structure or cap) and blood vessels, displaying a normal design. The study group showed a notable increase in necrotic tissue and cerebral blood vessel congestion. Neurons exhibited reduced cellular integrity, characterized by a lack of nuclear staining, a faintly discernible cell shape, and signs of softening and bleeding. Utilization of GCL in the context of SA reduced the occurrence of neuronal necrosis, neuronophagia, and neuronal edema. Mice treated with GCL alone exhibited a histological architecture within the normal range (Fig. 7A). Fig. 7B shows a photomicrograph of a mouse kidney in the control state. The image depicts the typical arrangement of renal corpuscles, consisting of glomeruli enclosed by proximal and distal renal tubules alongside a blood vessel. The image has been magnified 400 times (H&E 400×). The photomicrographs depict mouse kidney samples that underwent treatment with SA. A significant number of renal tubules exhibit apoptotic and necrotic changes, along with modest focal tubular vacuolization of the tubular cell cytoplasm, compared to the control kidney. Interstitial and intratubular hemorrhages are evident. The photomicrographs depict mouse kidney samples treated with SA and GCL. These images reveal the presence of localized interstitial inflammation characterized by diffuse and significant inflammatory infiltration. Furthermore, the photomicrographs also show the occurrence of apoptotic and necrotic renal tubules in the kidney tissue. The image provided is a photomicrograph of a mouse liver treated with GCL alone.

The image displays the typical structure of the renal corpuscles, including the glomeruli, as well as the proximal and distal renal tubules. In addition, the image depicts blood veins within the liver. As shown in Fig. 7C, liver samples from the control group were subjected to histological examination, which revealed a normal liver morphology without any signs of inflammatory cell infiltration or necrosis. The photomicrograph depicts a mouse liver segment subjected to SA treatment (Group II), revealing the presence of a dilated and clogged central vein. Hepatocyte necrosis accompanied by bleeding within the clogged central vein, portal vein, and hepatic sinusoids is also evident. Necrosis was predominantly observed in both the centrilobular and portal space areas. The necrotic hepatocytes exhibited barely identifiable nuclei, vacuolization, or cellular changes. Acute infiltration of inflammatory cells was observed around the portal area, in close proximity to the clogged central vein. Additionally, there was substantial foamy alteration in the Kupffer cells. The image provided is a photomicrograph displaying a liver segment from a mouse treated with SA and GCL, specifically belonging to Group III. In addition to the aforementioned findings, this group exhibited centrilobular necrosis, cellular edema, and vacuolar degeneration. Bleeding was also observed in the dilated portal veins. The observed inflammation surrounding the bile duct exhibited a notably decreased intensity. The portal tract included the portal vein, hepatic artery, and bile duct branches within its territory. Consequently, the administration of GCL resulted in a notable enhancement of the liver histopathology, as observed at a high-power magnification of 400× using H&E staining. The provided image is a photomicrograph displaying a segment of the mouse liver that was treated with GCL alone (Group VI). The image shows the typical structural arrangement of a healthy liver.

## 4. Discussion

Arsenic can cross the blood–brain barrier and is toxic to the neurological system [3,48]. In the present study, mice were subjected to SA exposure via the administration of the compound in their drinking water. The brain is susceptible to oxidative degradation due to increased oxygen consumption, substantial presence of polyunsaturated fatty acids, and deficiency in antioxidant defense mechanisms [49]. This study aimed to investigate the neuroprotective properties of GCL in mitigating redox imbalance and biomolecular changes related to the liver and kidney, and cognitive function in the brains of SA-treated mice. In our study, the Y-maze task was employed as a means of assessing short-term memory in both treated and untreated mice. Spatial alternation in the SA group was found to decrease the occurrence of spontaneous alternation behavior, which is indicative of a decline in memory function. Nevertheless, the administration of GCL had a positive impact on short-term memory, as evidenced by a notable increase in the proportion of alternating behavior in SA-treated mice. The MWM test was used to assess spatial working memory in SA-treated mice. Following the administration of SA, an extended period of time was required to escape, indicating the presence of memory impairment. Nevertheless, the administration of GCL resulted in decreased escape latency, indicating a potential enhancement in long-term and spatial working memory.

In the current study, we observed a correlation between arsenic exposure in mice and a reduction in AChE activity. The observed inhibition could be attributed to the direct or indirect suppression of both the central and peripheral cholinergic neurotransmitter systems. Furthermore, the observed inhibition could potentially arise from molecular interactions between arsenic and the sulfhydryl (-SH) group present in the enzymes. AChE is a crucial enzyme responsible for the rapid hydrolysis of acetylcholine, which is released at the neuromuscular junction. Under stressful situations, AChE activity is inhibited, thereby disrupting the breakdown of acetylcholine. In a previous study conducted by Agarwal et al., rats exposed to arsenic exhibited reduced AChE activity [50]. However, this decrease was partially mitigated by the administration of selenium, which has antioxidant properties [51]. Dwivedi et al. [52] have also shown a reduction in AChE activity following exposure to arsenic in rats.

Notably, pre-treatment with GCL significantly enhanced AChE activity. Previous studies have demonstrated that GCL can effectively scavenge free radicals and chelate metal ions, thereby inhibiting the interaction between the amyloid-beta peptide and the sulfhydryl (-SH) group of AChE. Nevertheless, it is worth noting that phenolic compounds resemble widely recognized AChE inhibitors, as highlighted by Oboh et al. [53]. A recent study showed that treating diabetic rats with curcumin resulted in reduced AChE activity, increased acetylcholine levels in the synapses, enhanced cholinergic neurotransmission, and improved antioxidant activities. Moreover, a potential association between ACE activity in the brain and neurological illnesses was identified by Labandeira-Garcia et al. [54]. The study conducted by Ali et al. (2023) revealed that patients diagnosed with Huntington’s disease and schizophrenia exhibited notably low levels of ACE activity [55]. Conversely, an increased expression of this enzyme has been detected in patients with Alzheimer’s disease (AD). The results of our study demonstrated a significant elevation in ACE activity in SA-administered mice. ACE activation initiates the enzymatic conversion of angiotensin I to angiotensin II. Therefore, an elevation in ACE activity may result in excessive production or build-up of angiotensin II within the central nervous system. However, the administration of GCL before and after treatment resulted in a significant reduction in ACE activity. GCL exhibited an inhibitory effect on ACE activity in SA-treated mice, which may have a protective role in mitigating oxidative damage to the brain and preventing inflammatory processes and neuronal impairment.

Assessment of liver cell integrity involves the estimation of AST and ALT enzyme levels. Research has indicated that elevated levels of AST and ALT in the plasma of rats subjected to arsenic exposure mostly result from impairment of the hepatic cell membrane. The damage leads to an increase in membrane permeability, which subsequently causes the leakage of cellular contents [56,57]. Based on earlier findings, a notable elevation in the levels of AST and ALT was detected in rats subjected to SA exposure compared to healthy control mice. GCL exhibited a potential inhibitory effect on ACE activity in SA-treated mice.

Several studies have documented a correlation between increased SA levels in the bloodstream and higher levels of serum urea and creatinine, as well as tubular damage [58,59]. Animal tests and epidemiologic studies conducted in humans have provided compelling evidence that both acute and chronic exposure to SA can result in kidney damage and an elevated risk of developing cancer [60]. Based on the findings of this investigation, it may be inferred that elevated levels of serum urea and serum creatinine observed after the 28-day treatment period indicate a heightened participation of renal tubular cells in the elimination of arsenic, rendering them susceptible to potential harm. Nevertheless, administration of GCL effectively mitigated the aberrant levels of blood urea and creatinine in the serum of mice. These findings indicate that GCL may have therapeutic benefits by enhancing purinergic transmission in the brains of SA-treated mice.

Enhanced LPO has been postulated to cause degenerative illnesses affecting the central nervous system. Oxidative degradation of polyunsaturated fatty acids by lipid peroxidation is a widely recognized consequence of arsenic exposure. In accordance, Fig. 3E-G illustrates that following the administration of SA for a duration of 1 week, a significant increase in LPO levels and a decrease in antioxidant levels occurred in the brains of mice. Elevated levels of LPO can be attributed to the collective impact of GSH levels, heightened production of free radicals, and suppression of antioxidant enzymes, such as SOD and CAT. Polyisoprenylated benzophenone compounds, which possess antioxidant properties, have the ability to operate as terminators of free radical chains and chelators of redox-active metal ions [27,29]. These metal ions accelerate lipid peroxidation. The findings of the current study align with this assertion, as pretreatment with GCL resulted in a decrease in LPO. Hence, we propose that GCL potentially mitigates the neurotoxic effects of SA by directly scavenging free radicals produced in the brains of mice after treatment with SA.

The signaling mechanisms responsible for the activation of HO-1 were further investigated to better understand its potential impact on the neuroprotective effect of GCL. The prevailing belief is that HO-1 serves as a target gene of Nrf2, a transcription factor that detects changes in cellular redox status and regulates the production of many antioxidant enzymes in response to oxidative stress [61]. Studies have demonstrated a notable decrease in the expression of HO-1 in mice lacking Nrf2, and the deletion of Nrf2 prevents the activation of the HO-1 gene. HO-1 enzymatically catalyzes the conversion of heme into biliverdin, free iron, and carbon monoxide [62,63]. HO-1 induces an upregulatory mechanism that effectively augments cellular resilience to various forms of stress. In addition, HO-1 plays a protective role in shielding microvascular cells from neurotoxicity generated by high glucose levels [64]. The present study revealed that administration of GCL increased HO-1 levels through the activation of Nrf2. Consequently, this leads to an augmentation in the functionality of antioxidant enzymes, including SOD and CAT, thereby impeding or averting the formation of ROS generated by SA.

The expression of genes encoding proteins that control critical brain processes, such as neuronal growth, learning, memory, metabolism, and neuroplasticity, is regulated by CREB [65]. Intracellular suppression of Gsk3β controls oxidative stress by specifically targeting neuronal Nrf2. According to existing evidence, inhibition of Gsk3β prevents neurotoxicity by enhancing the expression of genes related to oxidative stress. Geraniol has been found to have an inhibitory effect against Gsk3β. Therefore, it was postulated that it may have a neuroprotective effect against oxidative stress-induced neurotoxicity by interacting with the Gsk3β/Nrf2 signaling pathway, in addition to its direct antioxidant properties. In the present study, exposure to arsenic resulted in decreased expression of GSK3β and phosphorylation of CREB, while there was no significant change in total GSK3β and CREB. These changes are consistent with those reported by previous studies. Our research revealed that therapy using SA + GCL resulted in elevated levels of pCREB and pGSK3b. The concurrent administration of this therapy has anti-apoptotic and neuroprotective properties in murine brain tissue. The role of caspase-3 in neuroinflammation and apoptosis makes it a valuable therapeutic target for the treatment of neurodegeneration. The findings of this study indicated that treatment with SA resulted in a notable upregulation of expression of cleaved caspase and a concurrent decrease in Bcl-2 levels in the brain, as observed by Western blot analysis. The upregulated expression of caspase-3 in the brain tissue indicates the activation of this protease associated with the induction of neuroinflammatory pathways and apoptosis. This finding supports the previously observed degeneration of neurons in histological sections of the brain. In the experimental setting, the administration of SA to mice resulted in a decrease in caspase-3 levels and an increase in Bcl-2 expression, as shown by the analysis of GCL. Therefore, GCL has the potential to serve as an effective therapeutic agent, nutraceutical, or functional food for addressing cognitive impairments caused by arsenic exposure. However, further clinical trials are required to validate these findings.

## 5. Conclusion

To our knowledge, this is the first study to elucidate the molecular mechanisms underlying the neuroprotective effects of GCL in mice with arsenic-induced neurotoxicity. The results of our study indicate that GCL can modulate the levels of important biomolecules involved in cognitive function in the brains of SA-treated mice and that the administration of GCL may have a protective effect against cognitive dysfunction induced by treatment with SA. This protective effect is likely due to a reduction in oxidative damage, as evidenced by decreased MDA levels and caspase-3 expression. Additionally, GCL appears to modulate the activity of AChE and ACE, leading to improvements in cognitive behavior and prevention of neuronal degeneration in the brain. Therefore, it can be inferred that GCL is a potential therapeutic agent, nutraceutical, and/or functional food for addressing the cognitive impairment associated with AS. However, further clinical trials are required to validate these findings.

## Data Availability

All data included in this study are available upon request by contact with the corresponding author.
